# Analysis of T-DNA/Host-Plant DNA Junction Sequences in Single-Copy Transgenic Barley Lines

**DOI:** 10.3390/biology3010039

**Published:** 2014-01-21

**Authors:** Joanne G. Bartlett, Mark A. Smedley, Wendy A. Harwood

**Affiliations:** Department of Crop Genetics, John Innes Centre, Norwich Research Park, Norwich, NR4 7UH, UK; E-Mails: jgbartlett@gmail.com (J.G.B.); mark.smedley@jic.ac.uk (M.A.S.)

**Keywords:** transgene, flanking sequence, junction sequence, T-DNA integration, transgenic barley

## Abstract

Sequencing across the junction between an integrated transfer DNA (T-DNA) and a host plant genome provides two important pieces of information. The junctions themselves provide information regarding the proportion of T-DNA which has integrated into the host plant genome, whilst the transgene flanking sequences can be used to study the local genetic environment of the integrated transgene. In addition, this information is important in the safety assessment of GM crops and essential for GM traceability. In this study, a detailed analysis was carried out on the right-border T-DNA junction sequences of single-copy independent transgenic barley lines. T-DNA truncations at the right-border were found to be relatively common and affected 33.3% of the lines. In addition, 14.3% of lines had rearranged construct sequence after the right border break-point. An in depth analysis of the host-plant flanking sequences revealed that a significant proportion of the T-DNAs integrated into or close to known repetitive elements. However, this integration into repetitive DNA did not have a negative effect on transgene expression.

## 1. Introduction

Transgene junction sequence analysis (sequencing across the integrated T-DNA/host-plant DNA junction) can be used to examine the proportion of T-DNA which has integrated into a host plant genome, revealing whether or not the integration of plasmid backbone DNA has occurred, or whether the T-DNA region has become truncated or rearranged. The sequences flanking the T-DNA insertion enable the analysis of the transgene insertion site, revealing the presence of genic sequences or transposable elements. The data revealed by such analyses allows a more detailed understanding of transgene insertion as well as being of importance in the safety assessment of GM crops and in tracing individual GM events.

Flanking sequence analyses in barley have so far suggested a preference for the integration of transgenes into or close to genes, for both *Agrobacterium*-mediated transformation [1] and for particle bombardment transformation [2]. However only a small number of barley transgene flanking regions have been analysed so far. The observation that most transgenes appear to be located within coding sequences may be an artefact of the antibiotic selection procedure, whereby those lines which have transgenes integrated into non-expressing regions have such low expression that they are not recovered, and are hence not subject to analysis [3,4].

Significant alignments of host-plant flanking sequences to well-characterised sequences within the public databases can give an indication as to whether or not the transgene has integrated into or close to a native plant gene. The insertion of a transgene directly into a native plant gene can physically disrupt its expression, potentially affecting the phenotype of the plant. This mechanism has been exploited by researchers, with the creation of T-DNA insertional mutagenesis libraries. These libraries are providing a valuable resource for determining gene function in *Arabidopsis* [5,6,7]) and rice [8,9]. Determining the location of a T-DNA insertion via junction sequence analysis may therefore identify genes which have been disrupted, providing a tool for functional analysis, as well as enabling any effects of location upon gene expression to be observed.

Although it is generally assumed that the defined T-DNA of a plasmid will become integrated into the host plant genome, T-DNAs do not always excise from their originating plasmids at exactly the position of their right and left borders, and even when excised in the expected manner, T-DNAs can be subject to degradation or rearrangement within the plant cell prior to integration. When excision from the Ti plasmid occurs via a break within the T-DNA, or when the T-DNA becomes degraded within the plant cell, the integrated T-DNA is said to be truncated, and this truncation has the potential to reduce or prevent the expression of the transgene. Where additional plasmid sequence is incorporated due to a lack of cleavage at the left or right border, this additional plasmid “backbone” sequence is referred to as “read-through” and this can lead to unnecessary and/or unwanted gene sequences being integrated into the plant genome. 

In the study reported here, a detailed analysis was carried out on the right-border T-DNA junction sequences of 19 single-copy independent transgenic barley lines. Right-border T-DNA sequence (but not host-plant flanking sequence) was obtained for a further two lines, one showing evidence of rearrangement, and the other featuring the integration of construct backbone. An in depth analysis of the host-plant flanking sequences was used to determine whether or not transgenes preferentially integrate into or close to native genes. The relationship between transgene insertion site and transgene expression levels was also examined.

## 2. Experimental

### 2.1. Amplifying Junction Sequences

Transgenic barley DNA was obtained from randomly-selected single-copy transformed lines previously described by Bartlett *et al*. [10]. The DNA was taken from lines transformed with one of three different constructs, pBract215, pBract216 or pBract217. The pBract constructs are derivatives of pGreen [10]. The coding sequences of all three constructs were identical except that pBract216 and pBract217 featured an additional intron with the luciferase transgene. Of the 21 lines analysed, seven were transformed with pBract215, six with pBract216 and eight with pBract217. The DNA was extracted by the John Innes Centre Genome Laboratory. Leaf samples, each with a mass of approximately 50 mg (comprising of multiple small pieces), were submitted to the Genome Laboratory in 96-well plates and DNA was extracted from the leaf tissue using the Qiagen DNeasy 96 Plant kit (Q69181) system following the manufacturer’s instructions. 

**Figure 1:**
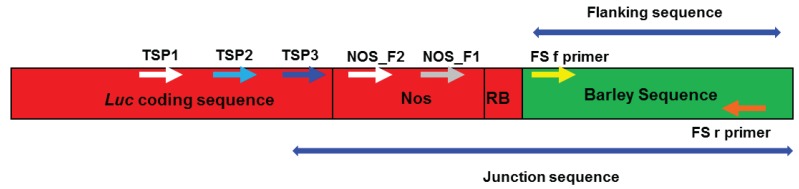
A diagram showing the approximate positions of the primer annealing sites (not to scale). Sequence derived from the transformation construct is shown in red, and barley genomic sequence is shown in green. The regions referred to as “junction sequence” and “flanking sequence” are labelled. The TSP1, TSP2 and TSP3 primers were used for junction sequence amplification. TSP3 was then used in combination with an FS_r primer to verify the junction sequence obtained for each line. The FS_f and FS_r primers were used in combination to amplify genomic DNA within untransformed Golden Promise, to confirm that the flanking sequence obtained derived from barley.

Junction sequences were obtained using the Seegene “DNA Walking *SpeedUp*^TM^ Premix Kit II” (Insight Biotechnology Ltd., Wembley, UK). The PCR methodologies followed the manufacturer’s instructions. Within these instructions, some of the parameters were flexible and could be optimised by the user. The specific parameters used in our experiments were as follows: PCR1 step 4 annealing temperature of 59 °C, PCR2 step 2 extension time of 100 s, PCR3 step 2 cycle number 30. The kit required the design of three nested target specific primers (TSPs). The following primers were designed for use with our transgenic lines: TSP1 (5'-GGATTACGTCGCCAGTCAAG-3'), TSP2 (5'-GTGTTTGTGGACGAAGTACCG-3') and TSP3 (5'-TCCTCATAAAGGCCAAGAAGG-3'). These were used in the first, second and third reactions respectively. They were designed to anneal towards the 3' end of the luciferase coding sequence, close to the T-DNA right border (Figure 1). Seven microlitres of DNA (with a concentration in the range of range of 15.8 ng/µL to 47.1 ng/µL) were used as template for the first PCR reaction. Two microlitres of purified PCR1 DNA (purified using a QIAGEN kit as recommended) were then used as template for the second PCR reaction and 1 µL of the second PCR reaction was used as template for the third PCR reaction. Twelve to fifteen microlitres of the third PCR reaction were run on a 1% agarose gel. Bands were excised from the gel, and DNA was extracted and purified using a QIAGEN QIAquick gel extraction kit (Cat. No. 28704).

### 2.2. Direct Sequencing

Sequencing was carried out using BigDye terminator v3.1 cycle sequencing. Dye-terminator reactions were composed of 1 µL BigDye v3.1, 1.5 µL 5× sequencing reaction buffer, 3.2 pmol sequencing primer and up to 6.86 µL gel-eluted template DNA made up to a total volume of 10 µL with H_2_O. Cycling parameters were as follows: 96 °C for 1 min, 96 °C for 10 s, 50 °C for 5 s, 60 °C for 4 min, return to step 2 25 times and 4 °C for 10 min. These reactions were then submitted to the John Innes Genome Laboratory for sequencing using an AbiPrism 3730 capillary sequencer. The TSP3 primer (see above) and the Seegene universal primer (UniP2: 5'-GAGTTTAGGTCCAGCGTCCGT-3'; provided with the DNA Walking *SpeedUp*^TM^ Premix Kit) were used for sequencing. Initially (for the first two batches of junction sequence analysis), estimates of DNA concentration (based on band brightness relative to a 100 ng band on the ladder, and taking into account the amount of DNA lost during gel elution) were combined with approximations of PCR product size, to determine how much DNA to add to the reaction (up to a maximum volume of 6.86 µL), according to quantities given by the BigDye v3.1 manufacturers. Volumes of DNA from 2 µL to 6.86 µL were added. Reactions in later batches of sequencing were all set up with either 5 or 6 µL template DNA so that the reactions could be prepared more efficiently, with the use of a master mix containing BigDye v3.1, buffer and H_2_O. Sequence traces obtained from the direct sequencing of PCR products were individually visualised, cropped (to remove poor quality sequence) and where necessary edited to correct errors. Where more than one sequence was obtained for one line (from sequencing with two primers and/or when more than one gel band was sequenced) these sequences were aligned to create a consensus sequence to be used in all further analysis.

### 2.3. Verification of Junction Sequences

Each junction sequence was verified using PCR, with the TSP3 primer (used for junction sequence amplification, see above) which anneals within *luc* and a reverse primer (referred to as FS_r, specific to each line) designed to anneal to the sequenced region of barley DNA. A list of the primers used for the verification process is given in Table 1 and a diagram showing the approximate positions of the annealing sites is given in Figure 1. Each initial verification PCR comprised 17 µL 1.1× ReddyMix^TM^ PCR Master Mix (ABgene), 1 µL of TSP3 primer (10 µM), 1 µL of line-specific FS_r primer (10 µM) and 1 µL of template DNA (template concentration in the range of range of 15.8 ng/µL to 47.1 ng/µL). Cycling parameters were 94 °C for 5 min, 94 °C for 30 s, 58 °C for 30 s, 72 °C for 55 s then return to step two 37 times. Gel electrophoresis of half of each reaction revealed that each PCR had generated a single amplified product. The remaining reaction (10 µL) was purified using ExoSAP-IT^®^ (GE Healthcare Life Sciences, Buckinghamshire, UK) according to manufacturer’s instructions. One microlitre of this reaction was then directly sequenced using BigDye terminator v3.1 cycle sequencing (outlined above) with the sequencing primer NOS_F1 (GCGCGGTGTCATCTATGTTA) or for line 87-09-01 (which has a truncated T-DNA) NOS_F2 (GCGCGCAAACTAGGATAAAT). 

**Table 1:** The primers designed to anneal within the T-DNA flanking sequences which were used for junction sequence verification. The corresponding amplicon product sizes are given for the different primer combinations used.

Line	FS_f primer (5' to 3')	FS_r primer (5' to 3')	TSP3 and FS_r amplicon size (bp)	FS_f and FS_r amplicon size (bp)
92-05-01	TGCATTTGCGGACTAATCAT	GAACAAGGGTGCGAAAGAAA	711	221
90-06-01	AACCCTTTCATCCGAACATC	GCCTGTTTACCGTCCGTCTA	656	270
84-13-01	CGTGTATGGTGTATACTAGCGTAAGA	GGGACGGGGTCTTTAGTTTC	536	170
84-15-01	GGCACGGTACAGTCCGTTTA	CACTCAAGCAGACCTGGACA	681	315
87-10-01	TCCCGTCAGTCAGTGAGATG	AGCAGGAGCCGATGAACG	811	252
88-06-01	TTGCCTACTTGCCTTGCTTT	TTGTTTCCCAATCACCACCT	810	241
88-13-01	CCTGCCAAACTGATCCAAAT	ACGGATTCACTGTCGCTGTC	778	220
89-02-01	GGGTGGATCTAGCGTACGAG	AGATCTGCACCGCATGAAG	N/A ^a^	247
89-07-01	TGTGCAAAGCAGTGTGTGAA	GATCGCATGCATGTACTCGT	770	281
85-01-01	GCATGCATTTCAGTGCTGTT	AGCTGCATGCTCCTGTTCTT	847	285
85-06-01	TGCCAGACCAGCTTTAATCA	CCTCTCAACAATGCCATGAA	957	354
86-03-01	ATCTCCCACTGATGCTCGAC	TCATGGATATGTCGCCTGTC	857	321
86-05-01	GCCTGTTGTTGGGAGTCG	GCCACTCCTTTCAGGAACTAAA	764	259
85-03-01	AGCCTGCGAGAATCTCTGGT	ACGATGCTCCATCATCATCA	807	296
91-02-01	CCACAAACCCTTACGCTATCA	GATGCTTCTGCGTGCAAGT	787	289
92-02-01	ATGAGCACCAATCATCACCA	CGCATGATTACGACAATCCA	818	294
90-04-01	GGTCATAATTAAACCCGCACT	CTAACGTGCATCGACTCCAA	598	238
71-09-01	AGCGAAGACGACAAGAGCAT	AAACAAAGGCGGTCAATGTG	784	294
83-06-01	AGGAAGTCGGAGCATAATTGA	AGGTAGGTATGGTGGCTGTTT	797	292

^a^ This line featured rearranged construct backbone sequence beyond the right border, therefore TSP3 could not be used to verify the junction sequence. A new primer, PCR_8902 (GCCTACATACCTCGCTCTGC) was used instead. This primer when used with FS_r gave an amplicon size of 816. The junction between the construct DNA and plant DNA for this line was sequenced using a new primer, Seq_8902 (GGGAAACGCCTGGTATCTTT).

The T-DNA/plant DNA junction sequences were first compared to the relevant construct sequence (either pBract215, pBract216 or pBract217 depending upon which plant line the DNA derived from), in order to determine the junction between the T-DNA and the native barley DNA. This was achieved using BLAST [11] and ClustalW2 [12]. Any sequence not identified to derive from construct DNA is referred to as T-DNA “flanking sequence” and is expected to be native barley DNA. For each flanking sequence, a forward PCR primer (referred to as FS_f) was designed to anneal close to the start of the barley DNA flanking sequence (see Table 1 and Figure 1). Each FS_f primer was used with the corresponding FS_r primer to amplify a 170–354 bp region of non-transformed Golden promise to confirm that the sequences adjacent to the integrated T-DNAs were of barley origin. The PCR mix and cycling parameters were identical to those described above for the initial verification PCR, except an FS_f primer was used instead of TSP3. 

The initial sequence obtained from line 84-14-01 (1,585 bp) was found have originated entirely from the transformation construct (T-DNA and sequential construct backbone). To investigate whether or not the entire backbone had integrated, the primers NpT1 (GCCTGAGCGAGACGAAATAC) and LB3 (ACGCGTCGAGTCTAGGTGAAGG) were designed to amplify a 1,400 bp region between this sequence, and the left-border of the construct.

### 2.4. Analysis of T-DNA Flanking Sequences

The barley T-DNA flanking sequences were first aligned to the TREP database [13] to look for regions of homology with known repeat elements. Sequences were then aligned to the non-redundant (nr), high throughput genomic sequences (htgs) and expressed sequence tag (est) NCBI databases using nucleotide BLAST (blastn) [11]. They were then aligned to the *Brachypodium distachyon* 4× assembly using the John Innes Centre BLAST server. 

## 3. Results

### 3.1. Right-Border Breakpoints

**Figure 2:**
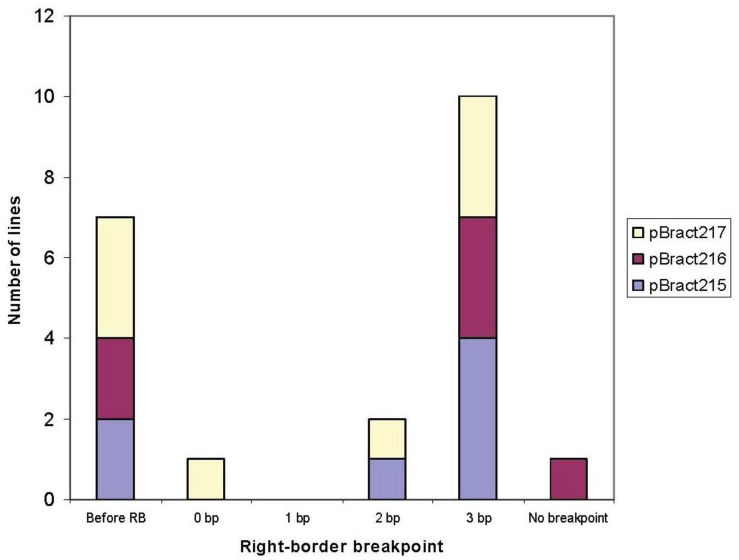
Right-border T-DNA breakpoints identified during junction-sequence analysis. The different colours correspond to the different constructs that the lines were transformed with.

Transgene junction sequences were obtained for nineteen independently-transformed single-copy lines. Right-border T-DNA sequence was obtained for a further two lines, however one of these sequences continued to read into the construct backbone and the other featured a right-border breakpoint followed by DNA from a different region of the construct. The “right-border breakpoint” can be defined as the position at which the integrated T-DNA sequence terminates. This position is determined by both the point of excision of the T-DNA strand and by the incidence of subsequent degradation within the plant cell. For the purposes of this study, we have assigned a number to each right-border breakpoint, representing the number of base pairs of sequenced construct DNA present within the plant genome beyond the start of the T-DNA 25 bp right-border repeat region. To clarify, if a T-DNA sequence is found to end 10 bases back from the start of the right border, it is given a breakpoint position of −10, and if 3 bases of the right-border repeat region are integrated into the plant genome, the breakpoint position of 3 is assigned. The right-border breakpoints and the amount of barley genomic flanking DNA obtained for each analysed line is given in Table 2. The breakpoint information is summarised in Figure 2. The intron composition of the transformation construct does not appear to be having any effect on the T-DNA breakpoint although it was shown to have a significant effect on transgene expression level [14].

Over 47% of analysed lines had a right-border breakpoint at three bases into the T-DNA right border repeat sequence. This was the most commonly identified right-border breakpoint and corresponds to the expected nick point between the 3rd and 4th nucleotide of the 25 bp repeat. No lines contained more than three base pairs of sequential construct DNA, apart from line 84-14-01, which did not contain a T-DNA breakpoint at the right border. In this line, it was found that the entire construct backbone sequence had integrated into the plant genome. Only three lines had a breakpoint between zero and two, and 33.3% of lines (7 out of 21) showed signs of truncation. The most highly truncated line (89-07-01) had a T-DNA sequence ending 81 base pairs back from the start of the right-border repeat sequence. The average level of truncation among the truncated lines was 24 bp. None of the truncated lines had their T-DNA breakpoints in the same position. Three lines featured unexpected construct sequence after the right-border breakpoint. These rearranged lines are summarised in Table 3.

**Table 2:** Right-border breakpoints and the amount of barley genomic flanking DNA obtained.

Construct	Line	Right-border breakpoint *	Barley flanking sequence obtained (bp)
pBract215	88-06-01	**3**	559
	88-13-01	**3**	877 (with 1 gap)
	85-01-01	**3**	1,050
	85-03-01	−7	632
	85-06-01	**3**	918
	91-02-01	**2**	417
	71-09-01	**−20**	1,007
pBract216	90-06-01	**3**	1,170
	84-13-01	**3**	178
	84-15-01	**−36**	627
	87-10-01	**3**	622
	90-04-01	**−10**	263
	84-14-01	None	0
pBract217	92-05-01	**−15**	1,551
	89-02-01	**0**	688
	89-07-01	**−81**	1,145 (with 1 gap)
	86-03-01	**3**	901
	86-05-01	**3**	1,092 (with 2 gaps)
	92-02-01	**2**	631
	83-06-01	**3**	448
	86-09-01	**−1**	0

* Right border breakpoint is defined as the number of sequential bases incorporated into the plant genome after the start of the right-border repeat region. A negative value indicates truncation of the T-DNA.

**Table 3:** Rearrangements present within the plant DNA at the T-DNA right-border.

Line	Construct	Right-border breakpoint	Barley sequence obtained (bp)	Additional DNA present after the right-border breakpoint
87-10-01	pBract216	3	622	167 bp of reverse-complement sequence from the left-border region. Sequence begins in the CaMv 35 s promoter (in the T-DNA) and ends 2 bp into the left-border repeat.
86-09-01	pBract217	−1	0	At least 315 bp of reverse-complement sequence from the left-border region, starting at the left-border repeat and going into backbone DNA.
89-02-01	pBract217	0	688	1,055 bp of reverse-complement backbone sequence from the right-border region. Construct sequence ends 2 bp into the right-border repeat from the 3' end

Two of the rearrangements were fully sequenced, and native barley sequence flanking the insertion site was obtained. Less sequence was obtained for the third line, so only part of the rearrangement was sequenced. All the additional sequences originate from either the left or right border region of the transformation construct, but for each line the rearrangements are different. For line 87-10-01, there appeared to be an overlap between the right-border and the additional sequence present after the breakpoint, with four base pairs of sequence potentially deriving from the end of the right-border or the beginning of the additional reverse-complement left border sequence. 

### 3.2. Analysis of Barley Flanking DNA

**Figure 3:**
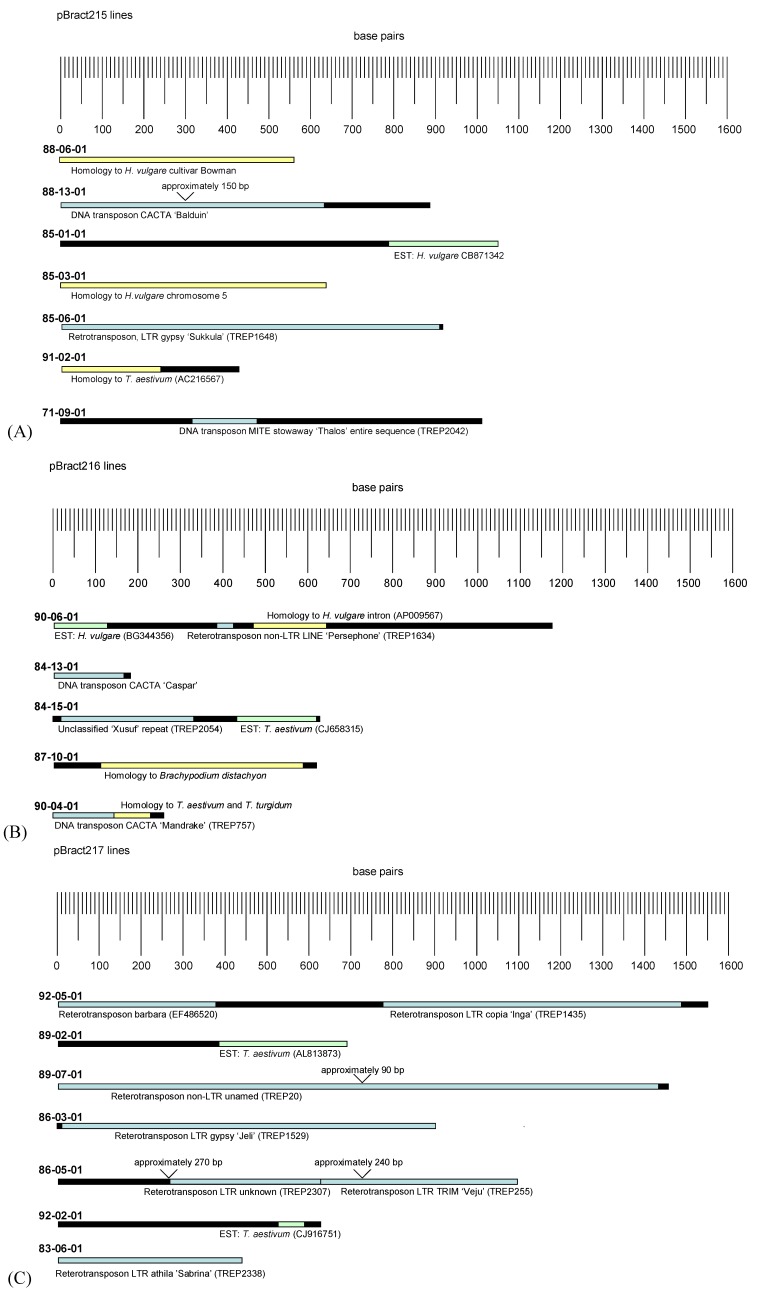
Alignments identified between T-DNA flanking DNA and sequences within the public databases. Flanking sequences for lines transformed with different constructs are given as follows: (**A**) lines containing pBract215, (**B**) lines containing pBract216, (**C**) lines containing pBract217. Blue sequences were homologous to known repetitive elements, green to ESTs and yellow to unannotated genomic DNA. Black sequences showed no significant homology to sequences within the public databases searched. Gaps in sequences (resulting from obtaining two or more non-overlapping sequences) are indicated, with approximate sizes given.

Primer pairs were designed to amplify a 170–354 bp amplicon within each of the flanking sequences and these were tested within Golden Promise. These PCRs confirmed that the flanking sequences were of barley origin. Barley transgene flanking DNA (with construct sequence removed) was separately aligned to sequences within a number of different public databases. Diagrams showing visual representations of the alignments are given in Figure 3. Two transgenic lines (88-06-01 and 85-03-01) initially showed no significant homology to sequences within the public databases searched. However, following the addition of barley genome sequence data [15], re-analysis of these sequences gave strong homology to regions of the barley genome. None of the flanking sequences showed regions of alignment to well-characterised plant genes. Over 60% of the flanking sequences characterised (12 out of a total of 19) contained regions of strong alignment (*e* value < 0.0001) to known repetitive elements. The majority of these elements were retrotransposons. Almost all of the retrotransposons identified showed evidence of transcription within barley or a related species, with 78% of the regions aligning to retrotransposons also aligning strongly to expressed sequence tags (ESTs) within the NCBI database.

Five alignments were made to ESTs which did not align to known retrotransposons (Figure 3). Four of these alignments were highly significant; with *e* values of less than 0.0001 (the *e* value of the 92-02-01 EST alignment was less significant). When the translated sequences were compared to proteins in the NCBI database, one (84-15-01) aligned to a rice hypothetical protein, and for the others there was no significant similarity. There was a further weaker EST alignment, with an e value of 0.18 (line 92-02-02). When translated, this EST also aligned to a rice hypothetical protein.

### 3.3. Relationship between Insertion Site and Transgene Expression

The lines for which junction sequence data had been obtained were further analysed to look for any patterns that might exist relating T-DNA insertion site or right-border (RB) breakpoint to T_0_ luciferase activity. This was undertaken to determine whether features of the insertion site might adversely impact transgene expression levels. Table 4 shows the lines for each construct arranged in order of increasing luciferase activity, with the junction sequence data given alongside. From the table, it is clear that no relationship between T-DNA insertion site and luciferase activity can be determined. Many of the lines appear to have their T-DNA inserted within a transposon or retrotransposon, but this does not seem to have affected transgene expression, as these lines exhibited a wide range of expression levels. There also appears to be no effect of RB breakpoint on luciferase activity.

## 4. Discussion

### 4.1. T-DNA Integration at the Right-Border

The T-DNA strand is excised from the transformation construct within the *Agrobacterium* cell, after the lower strand of the construct DNA is nicked at the right and left borders by the VirD1 and VirD2 proteins. T-DNA production is generally initiated at the right-border (at the 5' end of the strand), and terminated at the left border (the 3' end). However, there is also evidence of left-border initiated T-strand processing occurring [16,17].

**Table 4:** T-DNA insertion site compared to luciferase activity. For each construct the lines are listed in order of ascending luciferase activity. The flanking sequence features are summarised from those shown in Figure 3. The right-border (RB) breakpoint corresponds to the number of base pairs of sequential construct DNA present within the plant genome beyond the start of the RB repeat region.

Construct	Line	Luciferase activity (RLU/µg protein)	Flanking sequence features	Right-border breakpoint
pBract215	88-13-01	27028	Transposon directly adjacent to T-DNA	3
	85-01-01	28751	Barley EST 780 bp upstream of T-DNA	3
	88-06-01	39222	Homology to *H. Vulgare* cultivar Bowman contig	3
	85-06-01	64696	Retrotransposon directly adjacent to T-DNA	3
	85-03-01	65924	Homology to *H. vulgare* chromosome 5	−7
	71-09-01	93115	Alignment to transposon 310 bp upstream of T-DNA	−20
	91-02-01	96837	Homology to *T*. *aestivum* genomic sequence	2
pBract216	84-15-01	69628	Alignment to repeat element 20 bp upstream from T-DNA	−36
	90-04-01	80286	Transposon directly adjacent to T-DNA	−10
	90-06-01	98937	Barley EST directly adjacent to T-DNA	3
	84-13-01	107106	Transposon directly adjacent to T-DNA	3
	84-14-01	109189	No flanking sequence obtained	N/A
	87-10-01	115756	Region of homology to *B*. *distachyon*	3
pBract217	89-07-01	140921	Retrotransposon directly adjacent to T-DNA	−81
	89-02-01	278776	*T*. *aestivum* EST 384 bp upstream of T-DNA	0
	86-03-01	286903	Retrotransposon 16 bp upstream of T-DNA	3
	92-05-01	308998	Retrotransposon directly adjacent to T-DNA	−15
	86-09-01	312466	No flanking sequence obtained	−1
	86-05-01	364120	Retrotransposon about 500 bp upstream of T-DNA	3
	83-06-01	486226	Retrotransposon directly adjacent to T-DNA	3
	92-02-01	590787	*T*. *aestivum* EST 526 bp upstream of T-DNA	2

The right-border of the T-DNA is generally considered to be well conserved, with truncations deemed uncommon. The explanation for this is that the protein VirD2 binds to this end of the T-DNA strand to protect it against plant nucleolytic degradation [18]. This research however, shows that truncations can still commonly occur at this border, with 33.3% of the lines investigated here appearing to be truncated, losing between 1 and 81 bp of sequence prior to the start of the right border, with an average loss of 24 bp. None of the lines were truncated to the same extent, suggesting a more random process than mis-excision of the T-DNA at “pseudoborders” (non-border sequences that are falsely recognised as borders by *Agrobacterium*), a process previously proposed to explain truncations [19]. Possible alterative explanations for the truncations are occasional random nicking of the T-DNA (at non-border locations), nuclease digestion of the T-DNA ends prior to integration or breakage of the T-DNA during transfer. Herman *et al*., [20] found that the presence of the T-DNA right-border (within the binary plasmid) was required for the integration of truncated T-DNAs, ruling out the random nicking hypothesis, and suggesting that truncation occurs after the synthesis of a full length T-DNA, during the transfer or integration process.

As the study described here includes only single-copy luciferase-expressing lines, the analysis was biased towards lines containing intact copies of the luciferase gene. It is therefore possible that greater levels of truncation might have occurred in the non-expressing lines. Wu *et al*., [21] identified a small number of large T-DNA right-border truncations, however, a *GUS* gene that was positioned close to the right-border was conserved more frequently than a *bar* gene that was positioned close to the left border. In common with the present study, Gambino *et al*., [22] found several right border deletions in a study of T-DNA insertions in grapevine. In contrast, Zhang *et al*. [23] found that right borders were precise in cotton whereas most left borders had truncations.

The T-DNA appeared to be cleaved from the transformation constructs 3 bp into the right-border repeat region, with over 47% of lines (10 out of 21) having T-DNA breakpoints at this position, and no lines having additional sequential construct DNA after this point, apart from line 84-14-01 which lacks a right-border breakpoint. This supports the finding that nicking within the Ti plasmid occurs between the 3rd and 4th nucleotides of the right-border [24]. The absence of the right-border breakpoint in line 84-14-01 could indicate that the T-DNA was not nicked at the right border, suggesting that the initiation of T-strand synthesis proceeded from the left border, skipping the right-border, and leading to the entire construct sequence being integrated into the plant genome. Alternative mechanisms occurring during the process of integration, including truncations linked to micro homologies could also provide an explanation for the insertion of the entire backbone in this line.

The skipping of a termination border sequence (which can be the left or right border depending upon which initiated strand synthesis) has been proposed to be the result of a shortage of VirD2 molecules within the *Agrobacterium* cell, due to the presence of more copies of the binary plasmid than of the helper plasmid [25,26]. The integration of the full-length construct backbone has been reported to have occurred in a number of previous studies [17,21,25,26]). The presence of backbone sequence after the right-border that is not associated with “complete read through” (*i.e.*, the inclusion of the entire T-DNA backbone) has been found to be uncommon [20].

When producing commercial transgenic lines, it is important to identify all the DNA sequences that have been transferred to the plant. This can prove difficult when plasmid sequences are rearranged within the plant genome. Three of the 21 lines analysed here (14.3%) featured additional, rearranged sequences after what appeared to be the right-border breakpoint. The fragments of additional sequence were all different, but each one started or ended with left or right-border repeat sequence. These rearrangements may have occurred within the plant cell via interactions with other full length or fragmented T-DNAs within the same cell. Similar complex integration patterns were reported by Sha *et al*. [27] in transformed rice. However, few studies have reported T-DNA insertion events such as these, perhaps because in the majority of reports, truncation or read-through are measured via PCR and/or Southern analysis, without producing sequence of the precise T-DNA integration patterns. The data shown here highlights the need to fully sequence transgene junction sequences, in order to determine the exact composition of the T-DNA insertion site.

### 4.2. Analysis of Barley Flanking DNA

Twelve out of nineteen (63%) of the barley flanking regions aligned wholly or partly to repetitive elements, indicating insertion of the T-DNAs into repetitive regions of genomic DNA. As at least 60% of the barley genome is predicted to comprise of repeats [28], this is roughly the proportion of lines that would be expected to integrate into repetitive DNA by chance, if T-DNA integration occurred randomly throughout the genome. Zhao *et al*. [29] examined barley T-DNA flanking regions during the establishment of a gene tagging system using the maize Ds element. They found that 40% of the T-DNA insertions had significant homology to monocot EST database entries, around another 8% were close to coding regions and 36% of insertions were in repetitive regions suggesting a preference for insertion into non-redundant, gene-containing regions of the genome. However, when we took a subset (the first 21 lines) of these insertion sites and blasted against the TREP (Triticeae Repeat Sequence) database, 13 gave significant hits (from 75%–100% homology). This equates to 62% inserting into, or near to, repetitive elements, a very similar figure to that obtained in the current study. It is likely that the inclusion of alignments to the TREP database in the current study explains the increased proportion of flanking regions aligning to repetitive elements compared to that seen by Zhao *et al*. [29]. The majority of repeat elements identified in our study were retrotransposons, but some DNA transposons were also identified. Retrotransposons replicate by transcription followed by reverse transcription and integration of the cDNA back into the genome. Repetitive sequences within cereals are often referred to as being transcriptionally-inactive “junk DNA”, however recent studies have shown that many barley retrotransposons are active [28]. This finding is supported by the current analysis, with 77.8% of the retrotransposons identified here aligning to cereal EST sequences within the NCBI database. The presence of active repetitive regions within EST sequences provides a possible explanation for the apparent discrepancy between our findings and the findings of Zhao *et al*. [29]. Our findings are not in disagreement with the conclusion that T-DNAs may preferentially insert into active genomic regions. Previous studies in *Arabidopsis* [30,31]), rice [27], tobacco [20,30] and barley [2,29] have reported that transgenes have a tendency to insert into or close to genes. It is interesting therefore that such a high proportion of the single-copy T-DNAs characterised here appear to have inserted into or close to repetitive elements. However, the identification of one or more repetitive elements in a T-DNA flanking region does not necessarily mean that the T-DNA has not integrated into or close to a gene. Many repetitive elements are positioned close to genes, and the miniature inverted-repeat transposable element (MITE) for example (as identified in the flanking sequence of line 71-09-01) has a tendency to insert into the non-coding regions of genes [32]. Differences between this study and those carried out in *Arabidopsis* may also be the result of the differing genome compositions of *Arabidopsis* and cereal genomes. Whilst barley and other cereal genomes contain a high proportion of repetitive elements, only 5% of the *Arabidopsis* genome is predicted to comprise of repeats [28]. *Arabidopsis* therefore has a much higher gene density, making it more likely that T-DNAs will insert into or close to a gene by chance.

Some authors of previous reports have classified T-DNAs as being inserted into protein-coding regions solely on the basis of flanking sequence alignments to EST sequences [27] without attempting to align the flanking sequences or ESTs to characterised repeat elements. As shown by the analyses reported here this approach is unreliable, as many retrotransposons are represented in the EST databases. One may argue that an EST alignment at least confirms the presence of a T-DNA insertion close to a transcriptionally active region of the genome. However, similarity to a transcribed retrotransposon does not necessarily signify that the specific retrotransposon flanking the T-DNA is transcribed. Retrotransposons are present in high numbers within cereal genomes, and not all of them are active. 

Salvo-Garrido *et al*. [2] concluded that transgenes preferentially integrate into gene-rich regions of the barley genome on the basis of flanking sequence alignments obtained for seven lines generated by particle bombardment. Lines generated by particle bombardment present additional difficulties for the analysis of flanking sequences because the plasmid break-point must be identified before proceeding to isolate flanking sequences. In addition, such lines are known to contain more complex transgene integrations [33]. Physical mapping of transgene insertions suggested a non-random pattern of insertion [2]. This is in agreement with the analysis of large numbers of rice T-DNA flanking sequences that revealed a non-random distribution of T-DNA insertions with a bias towards certain chromosomes [34].

The flanking sequence data reported here appears to show no correlation between the local T-DNA integration-site and transgene expression. Previous reports have suggested that integration within repetitive DNA can lead to low transgene expression (of the gene of interest and the selection gene) which can bias the retrieval of transformed lines towards those with their T-DNA inserted within genic regions [4]. However, in this study, insertion of T-DNA into repetitive DNA did not appear to have an obvious negative effect on luciferase activity, with many highly expressing lines appearing to have their T-DNA inserted within retrotransposons. As noted before, individual retrotransposons may be active or inactive, and therefore it is not possible to conclude whether or not a particular T-DNA has inserted into a transcriptionally active region of genomic DNA purely on the basis of a retrotransposon or EST alignment.

We therefore propose that the evidence for preferential integration of transgenes into gene rich regions of barley is not as strong as previously reported. The data shown here suggests that the insertion of T-DNAs into repetitive regions of barley DNA is not having a negative effect on transgene expression, and hence is not leading to the biased selection of transformed lines with T-DNAs inserted within genes. A factor influencing this may be the high number of actively transcribed retrotransposons within the barley genome [28]. Of the 15 lines found here to be inserted into or close to regions aligning to repeats or ESTs, ten aligned strongly to repeats, three aligned only to ESTs, and two featured regions of alignment to both. A further four sequences showed no alignments to either although they did show homology to unannotated cereal DNA. The three ESTs which did not align to repetitive elements showed no significant alignments to characterised proteins; it is therefore possible that some of them correspond to further unidentified repeat elements.

It is now possible to target transgenes to specific genomic locations [35]. However, until such technology is used for all transgenic crop production, analysis of transgene flanking regions is required to fully understand the transgene genomic environment and to detect re-arrangements such as those highlighted in this study.

## 5. Conclusions

The safety assessment of GM crops demands the maximum amount of information on the transgene insertion. In the absence of precise targeting technologies to insert transgenes into specific genomic locations, analysis of the genomic environment surrounding transgenes, and of any rearrangements that may have occurred, remains crucial. This small-scale study of transgenic barley plants containing single copies of transgenes shows that DNA truncations and re-arrangements are relatively common at the right border of the T-DNA. In addition, a large proportion of the right border flanking sequences featured repetitive elements although this did not appear to have any effect on transgene expression levels. As additional sequence data is made available for key crops, and sequencing technologies continue to advance, the task of providing precise analyses of transgene insertion sites will become far more straightforward.
